# Reduced global longitudinal strain in association to increased left ventricular mass in patients with aortic valve stenosis and normal ejection fraction: a hybrid study combining echocardiography and magnetic resonance imaging

**DOI:** 10.1186/1476-7120-8-29

**Published:** 2010-07-26

**Authors:** Wilfried Dinh, Werner Nickl, Jan Smettan, Frank Kramer, Thomas Krahn, Thomas Scheffold, Michael Coll Barroso, Hilmar Brinkmann, Till Koehler, Mark Lankisch, Reiner Füth

**Affiliations:** 1 Institute for Heart and Circulation Research, University Witten/Herdecke, Germany; 2 Helios Clinics Wuppertal, Department of Cardiology, Wuppertal, Germany; 3 CoroVital, Institute for Sports Medicine, Wuppertal, Germany; 4 Global Biomarker Research, Bayer Schering Pharma AG, Wuppertal, Germany; 5 Department of Sports Medicine, University of Wuppertal, Germany

## Abstract

**Background:**

Increased muscle mass index of the left ventricle (LVMi) is an independent predictor for the development of symptoms in patients with asymptomatic aortic stenosis (AS). While the onset of clinical symptoms and left ventricular systolic dysfunction determines a poor prognosis, the standard echocardiographic evaluation of LV dysfunction, only based on measurements of the LV ejection fraction (EF), may be insufficient for an early assessment of imminent heart failure. Contrary, 2-dimensional speckle tracking (2DS) seems to be superior in detecting subtle changes in myocardial function. The aim of the study was to assess these LV function deteriorations with global longitudinal strain (GLS) analysis and the relations to LVMi in patients with AS and normal EF.

**Methods:**

50 patients with moderate to severe AS and 31 controls were enrolled. All patients underwent echocardiography, including 2DS imaging. LVMi measures were performed with magnetic resonance imaging in 38 patients with AS and indexed for body surface area.

**Results:**

The total group of patients with AST showed a GLS of -15,2 ± 3,6% while the control group reached -19,5 ± 2,7% (p < 0,001). By splitting the group with AS in normal, moderate and severe increased LVMi, the GLS was -17,0 ± 2,6%, -13,2 ± 3,8% and -12,4 ± 2,9%, respectively (p = 0,001), where LVMi and GLS showed a significant correlation (r = 0,6, p < 0,001).

**Conclusions:**

In conclusion, increased LVMi is reflected in abnormalities of GLS and the proportion of GLS impairment depends on the extent of LV hypertrophy. Therefore, simultaneous measurement of LVMi and GLS might be useful to identify patients at high risk for transition into heart failure who would benefit from aortic valve replacement irrespectively of LV EF.

## Background

The therapeutic management of patients with aortic stenosis (AS) depends on the severity of the stenosis and the presence of symptoms or the presence of left ventricular (LV) dysfunction, since the onset of symptoms and LV dysfunction determines a poor prognosis [1,2]. Particularly, patients with LV dysfunction show significantly worse outcome [3]. Notably, left ventricular dysfunction may develop insidiously in the asymptomatic patient.

The pathophysiological starting point in the development of subclinical LV dysfunction in AS is still a matter of debate. LV hypertrophy is a very common finding in AS and can result in early impairment in the LV performance. In order to compensate the elevated wall stress, the LV wall thickness increases, maintaining normal ejection fraction (EF) [4]. As there are two sides to every coin, LV hypertrophy is beneficial in some respects and harmful in other. In subjects with asymptomatic AS, increased left ventricular mass index (LVMi) was found to be an independent predictor for the development of symptoms in asymptomatic patients with severe AS [5]. Thus, the challenge for the clinician is to detect subtle LV contractile dysfunction at an early subclinical stage so that closer follow ups can be instituted or aortic valve replacement (AVR) can be performed to prevent irreversible LV deterioration. Recent guidelines only focus on LV EF to define systolic function. Nevertheless, since EF is based solely on endocardial radial motion, LV dysfunction may be underestimated by the standard parameter EF. Tissue Doppler imaging [6] and 2-dimensional strain (2DS) analysis of longitudinal myocardial function are superior in detecting subtle deteriorations of contractility [7]. In particular, analysis of global longitudinal strain (GLS) is a novel index for the assessment of global LV function and subtle deteriorations [8-10]. Hence, simultaneous measurement of LVMi and longitudinal myocardial function can provide new insights into the mechanical adaptation of the LV to chronic afterload elevation.

In the present study, we hypothesized that an increased LVMi in subjects with moderate to severe AS is reflected in abnormalities in GLS and that the proportion in GLS impairment depends on the extent of LV hypertrophy. In addition, we investigated whether GLS is sensitive enough to detect early recovery of global myocardial function after AVR.

## Methods

This study was planned for patients with moderate to severe AS who underwent conventional and 2 D speckle tracking echocardiography as part of clinical trial protocol. A total of 50 patients were enrolled. The study protocol was approved by the local ethics committee. Magnetic resonance imaging (MRI) was performed to assess LVMi and LV function. Exclusion criteria were concomitant mitral valve disease, severe low gradient AS, EF < 35%, hypertrophic obstructive cardiomyopathy, uncontrolled hypertension, severe ventricular arrhythmias, and general exclusion criteria for MRI.

Standard and tissue Doppler echocardiography were done with a commercially available system (Vingmed Vivid 7, General Electric, Milwaukee, Wisconsin). LV EF was calculated by the biplane Simpson's method. Left ventricular mass index was obtained by normalizing LVMi to body surface area according [11]. Doppler assessment of AS included the measurement of the peak and mean pressure gradient (Pmax and Pmean) and the transvalvular velocity (Vmax). Aortic valve area (AVA) was calculated by means of the continuity equation and indexed for body surface area (BSA); and pressure recovery adjusted aortic valve area (i.e. energy loss index, ELI) was calculated by a previously validated formula [12]. Severe AS was defined as AVA index < 0,6 cm^2^/m^2 ^with a Pmean ≥ 40 mmHg.

Deformation analysis of the datasets was performed off-line using EchoPac PC8.0 (General Electric-Vingmed). For this purpose, recordings of 3 consecutive 2 D images were used to analyze regional deformation on grey-scale images recorded from the parasternal LV short-axis (at the level of papillary muscles and LV apex) and apical four-, two- and three chamber views, respectively. The cardiac cycle with the best image quality and without any artefacts was selected for reporting results. Strain and strain rate analysis was performed as described previously [13]. In brief, the endocardial border was manually traced at an end-systolic frame. The software then automatically detected the frame-to-frame motion of the natural ultrasound reflecting markers (speckles). The position of myocardial speckles followed the longitudinal, radial and circumferential direction of motion. Aortic valve closure was identified from the continuous wave Doppler recording of the aortic valve flow. Results were reported as the peak during systole (peak systolic strain and peak systolic strain rate). Longitudinal measurements from the individual three apical standard views were averaged to obtain a GLS strain value [9]. The average peak radial systolic strain values were obtained from the parasternal short axis view at the level of the LV apex.

A 1.5-Tesla Achieva MRI scanner (Philips Medical Systems, Netherlands) equipped with a 5-element cardiac synergy coil was used. Cine-Images were acquired in breath hold SSFP sequences (TE 3.43, TR 1.72). Images were evaluated with the cmr42 research edition toolkit (circle cardiovascular imaging, Calgary, Canada) combining long and short axis views. The program calculated end- and endsystolic volumes, as well as stroke volume, ejection fraction and finally LVMi. Subjects were subsequently subdivided into three groups according the LVMi [11]: group 1 with normal LVMi, group 2 with mildly increased LVMi and group 3 with moderately or severely increased LVMi [11].

All analyses were performed using SPSS statistical software (SPSS 17.0, Chicago, IL). The data is presented as mean ± SD unless otherwise specified. An p < 0.05 was set considered statistically significant. Comparison of the 2 groups of subjects for various parameters was performed by 1-way analysis of variance (ANOVA). Pearson's linear correlation coefficients were calculated for pairs of continuous variables. We first analyzed associations without any adjustments and then with adjustments for potential confounders by multiple linear regression for continuous and logistic regression for categorical variables.

## Results

### Study population

50 patients with moderate to severe aortic stenosis and 31 controls without valvular heart disease and with normal EF were included. In 12 subjects with AS, either MRI or 2DS measurements was not performed because of bad image quality, low frame rate or claustrophobia. Therefore, both MRI measurements of LVMi and echocardiographic determination of GLS were done in 38 subjects. The baseline demographics and clinical characteristics are highlighted in table 1. Clinical characteristics did not differ between the different degrees of LVMi and controls. The presence of concomitant CAD was identified by angiography in 66% of patients with AS and in 45% of controls and was lower in patients with normal LVMi compared to those with mildly increased LVMi (p = 0,03), but not compared to those with moderately or severe increased LVMi (p = 0,85). The average logistic EuroSCORE was 11,1% and the additive EuroSCORE 7,2 in the study group, respectively.

**Table 1:** Demographics, clinical and laboratory characteristics

Aortic Stenosis vs. Controls				Aortic Stenosis			
**Variable**	**Aortic stenosis** **(n = 38)**	**Controls** **(n = 31)**	**p Value**	**Normal** **LVM** **(n = 21)**	**Mildly** **LVM ↑** **(n = 10)**	**Considerably LVM ↑** **(n = 7)**	**p Value**

Age (mean ± SD)	73 ± 9	69 ± 10	0,08	75 ± 5	70 ± 11	70 ± 15	0,31

Woman	42%	52%	0,29	48%	30%	42%	0,64

CAD	66%	45%	0,07	52%^#^	100%^#^	57%	0,03^#^*

Hx of MI	10%	36%	0,01*	5%	20%	14%	0,41

Hx of CABG	5%	10%	0,37	5%	0%	14%	0,42

Diabetes mellitus	34%	20%	0,15	27%	40%	43%	0,72

Hypertension	87%	92%	0,38	90%	80%	85%	0,72

Hyperlipidemia	53%	83%	0,01*	47%	60%	57%	0,8

Smoking	16%	57%	***0,001***	14%	10%	29%	0,56

ACE-inhibitor	66%	58%	0,34	62%	60%	85%	0,48

β-Blockers	76%	74%	0,52	81%	70%	71%	0,75

Statins	50%	64%	0,18	38%	50%	85%	0,41

CRP (mg/dl)	0,94	0,99	0,91	1,1	0,6	1,1	0,85

NTproBNP (pg/ml)		n.d.	n.d.	1635^#^	2015^#^	5988	0,05^#^*

Creatinine (mg/dl)	1,08	1,07	0,92	1,07	1,08	1,09	0,99

ACE = angiotensine converting enzyme, CAD = coronary artery disease, dl = deciliter; Hx = history of, LVM = left ventricular muscle mass index, mg = milligram, MI = myocardial infarction, n.d. = not done, SD= standard deviation, ↑ = increased, * = significant (p < 0,05)

### Echocardiographic and MRI measurements

**Figure 1:**
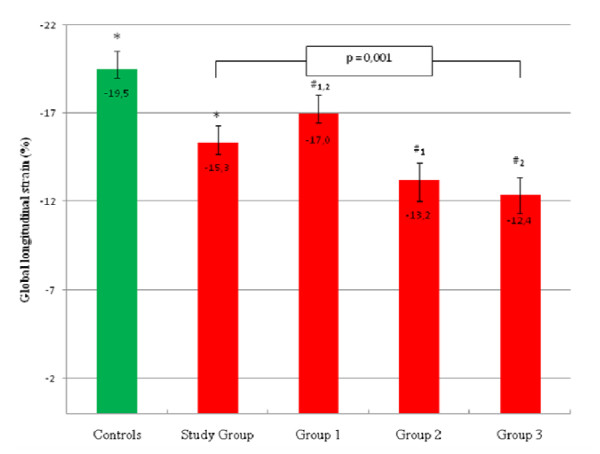
**GLS in relation to left ventricular mass index in controls and patients with aortic stenosis**. The range of average peak longitudinal strain in subjects with normal (group 1), mildly increased (group 2) and moderately to severe (group 3) increased LVMi and aortic stenosis, controls and the whole study group (n = 38). Left ventricular mass measurements indexed for body surface area were done with MRI. P = 0,001 for comparison between three groups by full-factorial Anova analysis of variance. #^1 ^P = 0,008 between average peak longitudinal strain in group 1 vs. group 2 and #^2 ^p = 0,004 between the group1 and group 2 by the Bonferroni post hoc test, respectively. *p < 0,001. GLS = global longitudinal strain, LVMi = left ventricular mass index.

**Figure 2:**
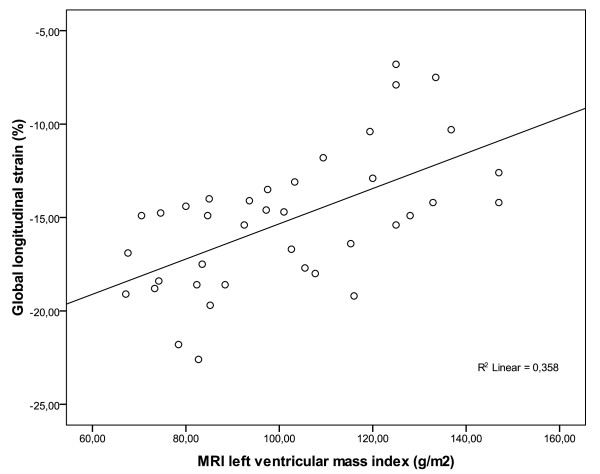
**Average global longitudinal strain (%) plotted against left ventricular mass index**. Average global longitudinal strain (%) plotted against left ventricular mass index shows a moderate, significant correlation (r = 0,6; p < 0,01). MRI = Magnetic resonance imaging.

Echocardiographic and MRI measurement results are summarized in Table 2. According to the indexed aortic valve area (AVA), 32 (84%) patients were classified as having severe aortic stenosis (AVAindex < 0,6 cm/m2), whereas 6 (16%) subjects were identified with moderate AS (AVAindex 0,6-0,85 cm/m2). Left ventricular muscle mass measurement reveals a normal LVMi in 64% of subjects with AS (group 1), whereas increased LVMi was detected in 44% of the study group: 26% with mildly increased LVMi (group 2) and 18% with moderately or severely increased LVMi (group 3). The echocardiographic image quality was sufficient to analyze longitudinal myocardial strain in 96% of patients included. In addition, GLS values were obtained in the control group. One-way ANOVA analysis demonstrated that the total group of patients with AST had significantly reduced GLS values (-15,2 ± 3,6%), compared to controls (-19,5 ± 2,7% p < 0,001). By splitting subjects with AS in group 1,2 or 3, the GLS was -17,0 ± 2,6%, -13,2 ± 3,8% and -12,4 ± 2,9%, respectively (Fig. 1, p = 0,001). The post-hoc analysis showed a significant difference between group 1 vs. group 2 (p = 0,008) and between group 1 and group 3 (p = 0,004) and LVMi and GLS correlated significantly (Fig. 2, r = 0,6, p < 0,001) in the whole study group.

**Table 2:** Echocardiographic and MRI measurements in subjects with aortic valve stenosis summarized.

Variable	All patients (n = 38)	LVM Normal (n = 21)	LVM mildly increased (n = 10)	LVM considerably increased (n = 7)	p- Value
LVM (MRI, g/m^2 ^BSA, SD)	101 ± 23	84 ± 12	116 ± 11	131 ± 14	

LVM (Echo, g/m^2 ^BSA, SD)	121 ± 36	103 ± 29	132 ± 31	161 ± 23	

GLS Baseline (%)	-15,2 ± 3,6	-17,0 ± 2,6	-13,2 ± 3,8	-12,4 ± 2,9	0,001*

GLS after AVR (%)	-17,6 ± 3,2	-19,5 ± 2,8	-15,4 ± 2,4	-15,8	0,04*

Vmax (cm/s)	434 ± 71	428 ± 72	426 ± 64	462 ± 80	0,51

Pmax (mmHg)	77 ± 26	75 ± 26	74 ± 22	87 ± 30	0,5

Pmean (mmHg)	45 ± 18	43 ± 17	43 ± 16	53 ± 22	0,37

AVA (cm^2^)	0,86 ± 0,23	0,89 ± 0,18	0,87 ± 0,26	0,73 ± 0,32	0,32

AVA index (cm^2^/m^2 ^BSA)	0,47 ± 0,12	0,49 ± 0,08	0,46 ± 0,12	0,42 ± 0,21	0,41

ELI (cm^2^/m^2 ^BSA)	0,54 ± 0,16	0,56 ± 0,11	0,53 ± 0,15	0,48 ± 0,28	0,49

E/A	1,1 ± 0,8	1,0 ± 0,5	1,1 ± 0,9	1,4 ± 1,3	0,53

Smax (cm/s)	4,8 ± 1,3	5,1 ± 1,3	4,7 ± 1,4	4,0 ± 1,0	0,14

E' (cm/s)	4,5 ± 1,2	4,9 ± 1,2	3,8 ± 1,2	4,5 ± 1,2	0,06

E/E'	20,5 ± 8,6	20,6 ± 8,8	21,5 ± 9,9	18,5 ± 6,5	0,78

CO (Echo, l/min)	5,0 ± 2,1	4,9 ± 2,3	5,3 ± 2,5	4,7 ± 0,9	0,85

SV (MRI, ml/min)	86 ± 22	84 ± 24	90 ± 23	85 ± 14	0,77

EF (Echo, %)	64 ± 12	67 ± 8	60 ± 15	61 ± 15	0,23

EF (MRI, %)	64 ± 10	68 ± 7	57 ± 12	63 ± 9	0,16

Left ventricular mass index was graduated based on MRI measurements.

A = late mitral inflow velocity; AVA = aortic valve area; AVR = aortic valve replacement; BSA = body surface area; CO = cardiac output, E = early mitral inflow velocity; E' = early tissue Doppler velocity at the septal mitral annuls, EF = ejection fraction; ELI = energy loss index; GLS = global longitudinal strain; LVM = left ventricular mass index; MRI = magnetic resonance imaging, P = pressure; S = systolic tissue Doppler velocity at the septal mitral annulus); SV = stroke volume; V = velocity

AVA, Pmean or EF did not differ between groups 1-3, respectively. In a multiple linear regression analysis including age, gender, hypertension, CAD, EF, history of myocardial infarction or history of coronary bypass surgery, AVAindex and Pmean, only LVMi remain a significant predictor variable for GLS impairment (Beta = 0,42, p = 0,009). Left ventricular EF (determined with MRI) and GLS (r = -0,42, p = 0,14) or LVMi (r = -0,30, p = 0,06) were not correlated.

The average peak radial systolic strain values (PRS) obtained from the parasternal short axis view at the level of the LV apex were tended to be higher in subjects with mildly increased LVMi compared to normal LVMi or moderately to severe increased LVMi (49,1 vs. 35,4 vs. 34,9%, p = 0,38).

**Figure 3:**
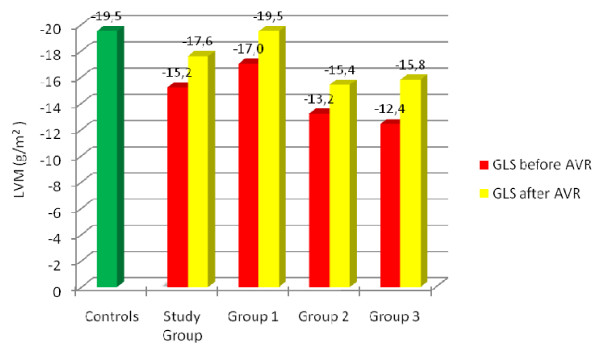
**GLS in relation to left ventricular mass index after aortic valve replacement**. The range of average peak longitudinal strain before and after AVR in subjects with normal (group 1), mildly abnormal (group 2) and moderately to severe (group 3) increased left ventricular mass index and aortic stenosis, controls and the whole study group (n = 38). GLS = peak longitudinal strain, AVR = Aortic valve replacement, LVM = left ventricular mass index.

A follow up visit at 3 month was done in 15 subjects. The average GLS values improved from -15,2 ± 3,6 to -17,6 ± 3,2%. All patients irrespectively of LVMi shows an augmentation of GLS (group 1 from -17,0 ± 2,6 to -19,5 ± 2,8%; group 2 from -13,2 ± 3,8 to -15,4 ± 2,4%; group 3 from -12,4 ± 2,9 to -15,8%, Fig. 3). Nevertheless, GLS was still reduced in subjects with mildly or moderately increased LVMi compared to controls (-19,5 vs. -15,4, p = 0,01).

## Discussion

To our knowledge, this is the first study to report a significant relationship between echocardiographic analysis of GLS and the degree of increased LVMi measured with MRI techniques. Our study adds several interesting findings adding to current knowledge on the pathophysiology of heart failure development in patients with AS.

Primarily, the extent of LV hypertrophy was independently associated with lower GLS, irrespectively of pressure gradients, coronary artery disease (CAD), EF or the severity of AS. In particular, in this study population, patients with more advantaged stages of LV hypertrophy had the lowest average GLS, demonstrating that this type of hypertrophy is characterized by low myocardial function. Secondly, impaired myocardial function can be detected by global myocardial longitudinal function assessment but not by LV EF measurements. Finally, GLS improved after AVR three month after surgery.

From the pathophysiological point of view, these observations are consistent with the concept that pressure overload results in extensive LV remodeling leading to hypertrophy, keeping wall stress normal. Since wall stress is a key determinant of ejection performance, its normalization is important in maintaining a normal EF. Because the EF is based solely on endocardial radial motion, the EF tends to increase relating to the extent of LV hypertrophy in early stages. In our study, radial strain, as a measurement of radial contraction [14], was preserved or even increased in subjects with mildly increased LVMi compared to those with normal LVMi or severely increased LVMi. Therefore, in early stages of LV hypertrophy, radial function acts as a compensatory phenomenon to the decrease in longitudinal deformation to maintain a normal LV ejection fraction [15].

Furthermore, it was shown that patients with LV hypertrophy have an increased incidence of cardiac events, including fatal ones [16]. Despite the fact that LV hypertrophy helps to preserve EF, it also impairs coronary blood flow reserve, which first occurs in the subendocardial layers and is associated with increased mortality [16,17]. It should be emphasized that the increase in wall stress and intramyocardial pressure as well as the consecutive reduction in myocardial blood flow occurs mainly in the subendocardium. The subendocardial myocardial fibers are oriented longitudinally. Thus, the selective impairment in longitudinal myocardial kinetics observed in our study might be due to the increase in subendocardial wall stress and associated ischemia and consecutive fibrosis. Hildick-Smith et al. showed in a previous study that coronary flow reserve increases after AVR, and this increase occurs in tandem with regression of LV hypertrophy [18].

Nevertheless, until now, the relation of GLS impairment to the extent of LV hypertrophy has not yet been clearly established. Echocardiographic imaging is the most widely available clinical tool to detect LV hypertrophy, as determined by the calculated LVMi. A major limitation of previous studies investigating the association between LVMi and GLS is the fact that the reproducibility of LVMi measurement by echocardiographic techniques is still controversial and prone to imaging artefacts. Therefore, MRI is a more precise and reliable method to quantify the mass of the left ventricle [6]. MRI is considered the ideal method for the determination of LVMi because of its high spatial resolution, generally good image quality, and ability to reconstruct the shape of the heart in three dimensions [19]. Therefore, we performed LVMi measurements with MRI [6] and GLS with 2 D echocardiography because of its higher temporal resolution and angle independency [13]. Combining the advantages of both methods, we believe that our results are robust with minor measurement artefacts.

Previous studies demonstrated that longitudinal strain measurement is superior to other indices of LV systolic function to predict symptoms, exercise tolerance and outcomes in AS patients [20,21]. Consequently, analysis of deformation in the longitudinal direction provides the most powerful approach to unmask subtle myocardial dysfunction that is not detected by EF in early stages. Even so, EF is the only index that is included in the recent guidelines to identify LV systolic dysfunction, a class I indication for AVR. In the SEAS trial [22], one third of asymptomatic patients with AS and preserved EF had a significant impairment of myocardial function. Hence, EF cannot exclude the presence of intrinsic myocardial dysfunction.

Additionally our study showed that GLS improved an average by - 2,5% 3 month after AVR, which previous studies confirmed as well [23,24]. After AVR, cardiac afterload decreases in relation to a decrease in LV pressure overload. Over time, the LV adapts to the new loading condition with a regression of hypertrophy and improvement of LV longitudinal strain. Notably, the EF did not show any significant changes [25]. Therefore, in the first instance, the longitudinal myocardial function improves [26]. Accordingly, 2DS assessment of GLS may provide a sensitive tool to detect improvement of myocardial function early after AVR.

In summary, our results demonstrate that the association between average GLS and LVMi is independent of age, CAD, severity of AS and LV EF. Because of the relationship between LV hypertrophy and subtle LV dysfunction, our findings may help to explain why concentric LV hypertrophy has been associated with higher in-hospital mortality after aortic valve replacement [27]. This is of practical importance since today's guidelines include only LV EF in management decisions in patients with AS [1]. These finding justifies the assessment of GLS in patients with AS because GLS helps to identify patients the transition from compensatory hypertrophy to myocardial failure.

### Study limitations

There are several limitations to our study. First, MRI determination of LVMi was not done in controls and either GLS or MRI was not possible in 12 subjects with AS. Nevertheless, the control group only serves to provide a basis for the measurements of GLS values in subjects without AS and comparable age and co-morbidities like CAD or hypertension. Furthermore, the study population was heterogeneous including subjects with or without concomitant coronary artery disease (CAD). However, CAD is a very frequent associated co-morbidity in AS and therefore our study population is reflecting clinical daily routine. Additionally, to date only 15 patients have been followed up, and LVMi was only measured with echocardiography at follow up visit. However, even statistically arguable, the overage improvement of GLS is consistent with reported literature and shows the appropriateness of 2DS analysis in the detection of subtle improvements early after AVR. Lastly, antihypertensive treatment was not standardized and left to the decision of the general practitioners managing the individual patients. Thus, impact of antihypertensive treatment on deformation parameters could not be assessed in the study.

## Conclusions

Our findings provide new insights into the mechanical adaptation of the LV to chronic afterload elevation and its response to unloading. In patients with AS, increased LVMi is associated with a progressive reduction in GLS assessed by 2DS. The degree of hypertrophy parallels the severity of overload, and GLS improves after AVR. However, left ventricular hypertrophy does not necessarily imply myocardial dysfunction. Therefore, assessment of GLS can identify subtle contractile dysfunction triggered by increased LV mass and might be useful to identify patients at high risk who would benefit from AVR. The prognostic implications of our findings remain to be assessed in future longitudinal studies.

## Competing interests

The authors declare that they have no competing interests.

## Authors' contributions

WD conceived of the study, participated in the study and drafted the manuscript and performed statistical analysis. WN participated in the study and drafted the manuscript and performed statistical analysis. JS performed MRI analysis. FK helped to draft the manuscript. TS participated in the study design of the study and performed statistical analysis. MC-B participated in echocardiographic studies. HB performed MRI analysis. TK participated in echocardiographic studies and helped to draft the manuscript. ML participated in the study design and coordination and helped to draft the manuscript. RF participated in echocardiographic studies and participated in the study design.

All authors have read and approved the final manuscript.
